# The Clinical Values of Afamin, Triglyceride and PLR in Predicting Risk of Gestational Diabetes During Early Pregnancy

**DOI:** 10.3389/fendo.2021.723650

**Published:** 2021-11-03

**Authors:** Xuechun Wang, Xiuqiong Zheng, Jianying Yan, Rongli Xu, Mu Xu, Lin Zheng, Liangpu Xu, Zhi Lin

**Affiliations:** ^1^ Department of Obstetrics, Fujian Maternity and Child Health Hospital, Affiliated Hospital of Fujian Medical University, Fuzhou, China; ^2^ Fujian Key Laboratory for Prenatal Diagnosis and Birth Defect, Fujian Maternity and Child Health Hospital, Affiliated Hospital of Fujian Medical University, Fuzhou, China

**Keywords:** gestational diabetes, early prediction, afamin, age, PLR, triglycerides

## Abstract

**Objective:**

To establish a model to predict gestational diabetes mellitus (GDM) based on the clinical characteristics, early pregnancy (10-12 weeks gestation) peripheral blood routine, and biochemical indicators, and to explore its predictive efficiencies.

**Methods:**

Data from 607 pregnant women with GDM were compared to the data from 833 pregnant women without GDM admitted to the Obstetrics Department of Fujian Maternity and Child Health Hospital (affiliated to Fujian Medical University) from May 2018 to December 2018 were retrospectively included. The ages of the pregnant women, paternal ages, number of pregnancies, number of deliveries, pre-pregnancy heights/weights, and the calculated body mass indexes (BMI) were recorded. In all participants, 10-12 weeks of pregnancy, afamin concentration, routine blood work, prenatal aneuploidy screening, and biochemical testing were performed. At weeks 24-28 of gestation, patients underwent oral glucose tolerance test (OGTT) for GDM screening.

**Results:**

Multivariate logistic regression analysis showed that maternal age, early pregnancy afamin level, triglycerides, and platelet/lymphocyte ratio (PLR) were independent risk factors for gestational diabetes. The formula for predicting GDM probability was as follows: *P* = 1/1 + *exp*( − 6.054 + 0.774 × *triglycerides* + 0.002 × *afamin* + 0.155 × *age* − 0.012 × *PLR*)]. From the established ROC curve, the area under the curve (AUC) was 0.748, indicating that the model has a good degree of discrimination. When the predictive probability cut-off value was set on 0.358, sensitivity, specificity, positive predictive value, and negative predictive value were 69.2%, 68.3%, 42.5%, and 86.2%, respectively, and the accuracy rate was 70.2%. The Hosmer-Lemeshow test results showed that the goodness of the model fit has a good calibration ability (χ2 = 12.269, df=8, P=0.140).

**Conclusions:**

Maternal age, early pregnancy afamin level, triglycerides, and PLR are independent risk factors for gestational diabetes. When combined, the above indicators are helpful for prediction, early diagnosis, and intervention of gestational diabetes.

## Introduction

Gestational diabetes mellitus (GDM) refers to diabetes that often starts in the middle or late stages of pregnancy and is considered one of the most common diseases during pregnancy (1). According to the diagnostic criteria from the International Association of Diabetes and Pregnancy Study Group (IADPSG), the incidence rate of GDM is about 13.9% (2). Recent years, with the prevalence of obesity, delayed childbirth, multiple pregnancies, and these factors, there is a gradual increase in GDM occurrence, with a greater impacts to the wellbeing of both mother and baby (3). Gestational diabetes mellitus can increase the incidence of miscarriage, premature delivery, dystocia, infection, pregnancy complications, fetal macrosomia, structural birth defects, and small for gestational age babies (4). Gestational diabetes mellitus also significantly increases predisposition to non-GDM diabetes after pregnancy (5). Since blood glucose screening is usually performed in the middle and late stages of pregnancy, once confirmed, there is little time for patients to receive treatment and prevent the disease from negatively impacting the fetus. Therefore, there is a need to identify some appropriate biochemical markers for the early diagnosis of GDM. Afamin, a vitamin E–binding protein, seems to play a role in anti-apoptotic cellular response to oxidative stress (6). Studies indicate that afamin plasma levels increase linearly (around 2-fold) during an uncomplicated pregnancy (7), and strongly correlate with clinical and laboratory parameters of the metabolic syndrome, such as elevations in BMI and plasma glucose concentrations (8). Recent large population-based study in more than 20,000 participants showed strong association of afamin concentrations with the prevalence and incidence of type 2 diabetes mellitus (9), making it a potential new biomarker for pathological glucose metabolism in pregnancy. The main goal of this retrospective study is to conduct in-depth research of GDM to find new accurate and reliable predictive methods of diagnostics.

## Materials and Methods

### Research Participants and Groupings

Medical data from pregnant women who gave birth in our hospital from May 2018 to December 2018 were retrieved from the Medical record of our hospital and retrospectively grouped according to glucose tolerance test results of the patients at 24-28 weeks of gestation.

Based on the recorded results of the glucose tolerance tests, 607 cases were included in the GDM group, and 833 pregnant women with normal glucose tolerance, matched according to the similar blood sampling time in the first trimester, were included in the non-GDM group. For all the participants, age of the pregnant woman, age of the father, number of pregnancies, parity, height/weight before pregnancy, and the calculated BMI before pregnancy were recorded.

**Figure 1:**
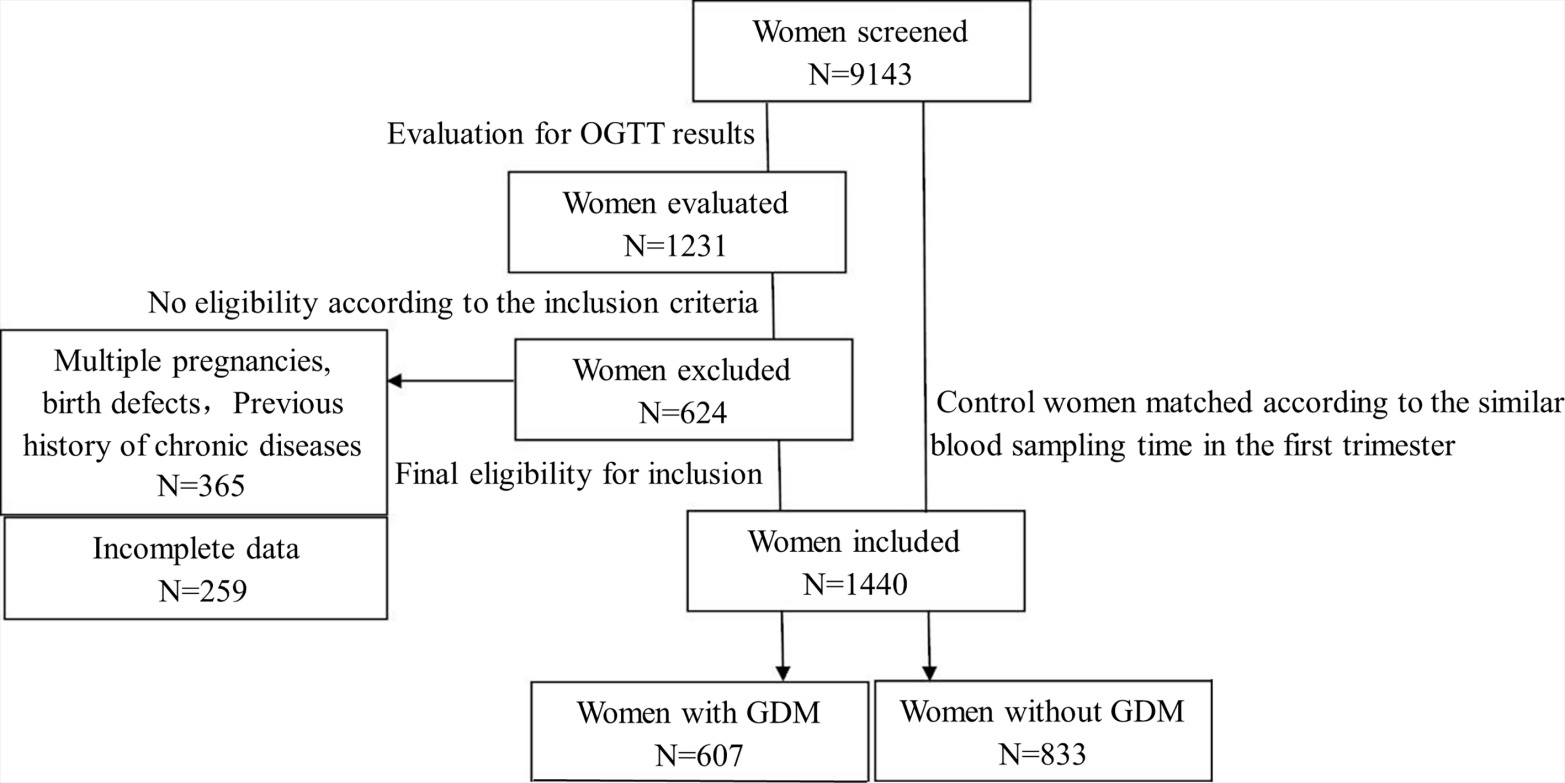
Schematic study workflow.

Gestational diabetes was diagnosed based on the diagnostic criteria established by the American Diabetes Association (ADA) in 2012 (10). Inclusion criteria were as follows (1): Singleton pregnancy (2); afamin blood test and routine blood test, prenatal aneuploidy screening (a combination of nuchal translucency thickness measurement, and levels of serum-free human chorionic gonadotropin and the pregnancy-associated plasma protein A), and biochemical tests performed and recorded at 10-12 weeks of gestation, combined with OGTT tests at 24-28 weeks of gestation (3); The data is complete and available. Exclusion criteria included: (1) Previous history of chronic diseases such as hypertension, diabetes, chronic liver or kidney disease, malignant tumor, autoimmune disease, blood system disease or certain infectious diseases; (2) Multiple pregnancy, birth defects, or miscarriage; (3) Incomplete or unavailable data. All participants in this study signed a consent form, and the research protocol was approved by the Ethics Committee of Fujian Maternity and Child Health Hospital (No: 2018-140) from June 12 2018. **Figure 1** summarizes a study flowchart.

### Afamin Level Tests

Two milliliters of fasting venous blood were drawn from all the included study participants in the first trimester (10-12 weeks of gestation). The samples were centrifuged at 4°C, 3,000 rpm for 30 minutes. The supernatant was extracted and stored at -80°C. Afamin concentration in the serum was measured by ELISA, using specific mono- and polyclonal antibodies recognizing human afamin (Shanghai, Huiying, China) according to manufacturer’s instructions.

Routine blood tests, screening for the Downs syndrome, and biochemical analysis were performed and recorded in the laboratory of our hospital. The parameters included afamin, white blood cell count (WBC), neutrophil count (NEU), platelet count (PLT), mean platelet volume (MPV), platelet count test (PCT), platelet distribution width (PDW), platelet large cell ratio (PLCR), lymphocyte count (LYM), hemoglobin (HGB), pregnancy-associated plasma protein A, triglyceride (TG), total cholesterol (CHOL), high density lipoprotein (HDLC), low density lipoprotein (LDL), serum ferritin (Ferr), Calcium (CA), and magnesium (MG). In addition, the neutrophil/lymphocyte ratio (NLR) and platelet/lymphocyte ratio (PLR) were calculated and recorded. NLR is defined as the ratio between the absolute value of neutrophil count and the absolute value of lymphocyte count, and PLR is defined as the ratio between the absolute value of platelet count and the absolute value of lymphocyte count.

### Statistical Analysis

SPSS 24.0 software was used for statistical analysis. Data were expressed as mean ± standard deviation, and independent sample t-test was used for comparison between groups. Variables with P < 0.05 in univariate analysis were further analyzed using multivariate logistic regression to identify independent risk factors for GDM. SPSS’s histogram chart was used to investigate the normal distribution of the data. When evaluating the specificity and sensitivity of certain parameter as a potential diagnostic prediction factor, receiver operating characteristic curve (ROC) and area under the curve (AUC) methods were used, and Youden index was employed to select the most appropriate cut-off value. We consider P<0.05 as a statistically significant value.

## Results

### Clinical and Laboratory Characteristics of Pregnant Women

There was no statistically significant difference between the two groups of pregnant women in pre-pregnancy BMI, number of pregnancies, and parity (P>0.05). There was a statistically significant difference between the study and the GDM groups in terms of maternal age (N (SD) (30.39 (3.30) and 28.42 (3.30) respectively; P<0.001). Similarly, the paternal age was significantly higher in the non-GDM group as compared to the GDM group (P<0.001). The results are summarized in **Table 1**.

**Table 1:** Comparison of clinical characteristics and laboratory indicators between the two groups of pregnant women.

Project	GDM group (n = 607 cases)	Non-GDM Group (n = 833 cases)	*P value*
Maternal age (years)	30.39 ± 3.66	28.42 ± 3.30	<0.001
Husband age (years)	31.80 ± 4.55	30.23 ± 4.03	<0.001
Number of pregnancies	2.05 ± 1.19	1.87 ± 1.04	0.004
Number of births (Times)	1.45 ± 0.57	1.41 ± 0.65	0.202
Pre-pregnancy BMI index(Kg/m^2^)	22.26 ± 7.31	21.67 ± 2.72	0.075
Afamin (ng/ml)	663.42 ± 226.13	594.26 ± 143.80	<0.001
PAPPA (mg/L)	2205.50 ± 1880.49	2563.45 ± 2066.32	0.001
MPV (fL)	10.00 ± 0.80	10.09 ± 0.91	0.063
PLT (×10^3^/L)	244.63 ± 47.95	237.47 ± 51.50	0.008
PCT (%)	0.24 ± 0.04	0.24 ± 0.05	0.024
PDW (%)	11.18 ± 1.68	11.31 ± 1.93	0.194
PLCR (%)	24.71 ± 6.54	25.34 ± 7.37	0.102
WBC (/uL)	8.98 ± 1.96	8.33 ± 1.86	<0.001
NEU (/uL)	7.01 ± 6.94	5.92 ± 3.52	0.001
LYM (/uL)	2.16 ± 2.02	1.96 ± 0.83	0.036
HGB (g/L)	127.76 ± 9.42	126.40 ± 9.86	0.009
NLR	3.45 ± 2.37	3.19 ± 1.72	0.011
PLR	113.98 ± 34.53	128.63 ± 37.51	<0.001
TG (mmol/L)	1.56 ± 0.73	1.31 ± 0.47	<0.001
CHOL (mmol/L)	4.65 ± 0.76	4.49 ± 0.69	<0.001
HDLC (mmol/L)	1.72 ± 0.32	1.71 ± 0.32	0.698
LDL (mmol/L)	2.09 ± 0.58	1.97 ± 0.52	<0.001
CA (mmol/L)	2.33 ± 0.81	2.33 ± 0.81	0.954
MG (mmol/L)	0.88 ± 0.70	0.89 ± 0.73	0.088
Ferr (μg/L)	84.61 ± 58.83	87.79 ± 65.18	0.404

GDM, Gestational Diabetes Mellitus; BMI, body mass index; WBC, white blood cells; NEU, neutrophils; PLT, platelets; MPV, mean platelet volume; PCT, platelet count test; PDW, platelet distribution width; PLCR, platelet large cell ratio; LYM, lymphocytes; HGB, hemoglobin; PAPPA, pregnancy-associated plasma protein A; TG, triglycerides; CHOL, total cholesterol; HDLC, high density lipoprotein; LDL, low density lipoprotein; Ferr, serum ferritin; CA, Calcium; MG, magnesium, (MG); NLR, neutrophil/lymphocyte ratio; PLR, platelet/lymphocyte ratio.

Serum afamin levels were significantly elevated in the women, diagnosed with GDM, compared to GDM group (P<0.001), **Table 1**. Similarly, parameters, such as WBC, PLT, NEU, LYM, HGB, NLR, TG, CHOL and LDL, were significantly elevated, while levels of PAPPA and PLR were markedly lower in non-GDM group as compared to GDM group (P<0.05). There was no statistically significant difference in MPV, PDW, PLCR, HDLC, CA, MG and serum ferritin between the two groups of pregnant women (P>0.05), **Table 1**.

### Multivariate Logistic Regression Analysis and Predictive Model Construction

We next carried out multivariant logistic regression analysis to identify possible risk factors of GDM. As summarized in **Table 2**, age, early pregnancy maternal blood platelet/lymphocyte ratio, triglycerides and afamin are found to be independent risk factors for GDM (P<0.001). The formula for predicting GDM probability was as follows:


P=1/[1+exp(-6.054+0.774×triglycerides+0.002×afamin+0.155×age-0.012×PLR)].

**Table 2:** Risk factors associated with gestational diabetes mellitus: multi-factor logistic regression analysis.

Index	OR value	(95% confidence interval)	*P value*
Age (years)	1.167	1.123,1.213	<0.001
Afamin(ng/ml)	1.002	1.001,1003	<0.001
TG (mmol/L)	2.168	1.674,2.809	<0.001
PLR	0.988	0.984,0.992	<0.001

TG, triglycerides; PLR, platelet/lymphocyte ratio;OR, odds ratio.

### Evaluation of the Prediction Model

**Figure 2:**
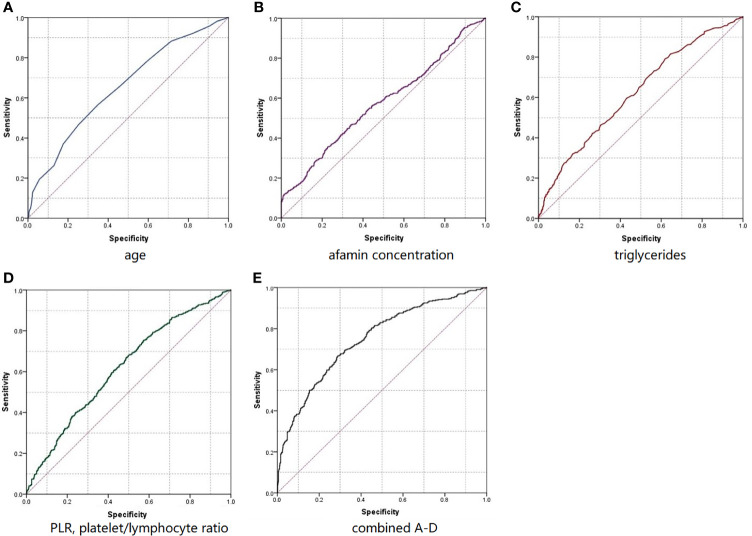
RoC curves predicted individually **(A–D)** or combined **(E)** using age **(A)**, afamin concentration **(B)**, triglyceride **(C)**, and platelet lymphocyte ratio **(D)** levels in the early stages of pregnancy.

We next evaluated the performance of a classification model using ROC and the area under the curve (AUC) metrics. As summarized in **Figure 2** and **Table 3**, AUC for age, afamin levels, TG, and PLR were 0.653, 0.574, 0.622, and 0.618, respectively. The AUC of the ROC curve established for the prediction model was 0.748, greater than the AUC of any single factor. The discrimination of the prediction model was, therefore, satisfactory. When the prediction probability cut-off value was 0.358, the sensitivity, specificity, positive predictive value, and negative predictive value for age, afamin levels, TG, and PLR were 69.2%, 68.3%, 42.5%, and 86.2%, respectively, with the accuracy rate of 70.2%. The Hosmer-Leme show test results showed that the model has a good discrimination ability (χ2 = 12.269, df=8, P=0.140), **Table 3** and **Figure 2**.

**Table 3:** Risk factors associated with gestational diabetes mellitus: predictive capabilities.

Index	Truncation value	Sensitivity	Specificity	AUC value (95% confidence interval)	*P Value*
Age (years)	29.50	56.6	65.3	0.653 (0.624,0.682)	<0.001
Afamin (ng/ml)	720.10	35.6	77.3	0.574 (0.543,0.605)	<0.001
TG (mmol/L)	1.095	79.1	38.4	0.622 (0.591,0.653)	<0.001
PLR	124.56	68.0	50.2	0.618 (0.588, 0.648)	<0.001
The predictive model	0.358	69.2	68.3	0.748 (0.719, 0.777)	<0.001

TG, triglycerides; PLR, platelet/lymphocyte ratio; AUC, area under curve.

## Discussion

Most patients with GDM will return to normal blood glucose levels shortly after delivery. However, GDM diagnosis carries a up to a 60% lifetime risk of progression to type 2 diabetes mellitus (T2DM) (11). GDM and T2DM share many common risk factors and pathological pathways. Studies showed that GDM women with severe form of blood glucose disorders during pregnancy generally progress to T2DM within 6 months to 5 years postpartum. Over time, the cumulative incidence of postpartum diabetes gradually increases (12, 13), indicating that GDM may be the early stage of T2DM (14). International Diabetes Foundation predicts that by 2040, the total number of diabetic patients in the world will reach 642 million (15). Therefore, early diagnosis and treatment of GDM can greatly reduce the incidence of maternal and infant complications, improve the prognosis of the disease, and also reduce the burden on healthcare system. Previous studies have shown that appropriate physical activity before and during pregnancy can reduce the risk of GDM (16). Another study confirmed that GDM screening in early pregnancy can effectively reduce the risk of GDM and fetal macrosomia. This indicates that control of blood sugar through diet and exercise in response to GDM screening can yield positive outcomes (17). With the liberalization of the second-child policy in China, more and more families choose to have second baby that leads to more advanced age pregnancies, contributing to the significant increase in the incidence of GDM (18). Early screening for the occurrence of GDM can, therefore, not only reduce the incidence of GDM and its negative outcomes, but also help to conform to the national advocacy of “eugenics” in China (19, 20). From both individual family’s welfare and national policy point of view, finding indicators to screen for early GDM, provide early prevention measures, give early diagnostic approaches, and implement early treatment methods have profound clinical application values.

The results of this study confirms previous reports that age is an independent risk factor for the onset of GDM (21). At present, there is relatively little controversy over the fact that age is an independent risk factor for the onset of GDM. Aging induces the overall physiological function declination of women, and insulin affinity also decreases with aging (22). The metabolism of various system organs increases during pregnancy to meet the needs of fetal growth and development. As result, compensatory insulin secretion increases, which in turn aggravates insulin resistance, potentially leading to development of GDM (23, 24).

Afamin, secreted mainly by liver, is also known as vitamin E binding protein, it a glycoprotein with multiple biological effects, including immune and inflammatory response (25). Recent studies showed that afamin can bind to thioredoxin, leading to oxidative stress and, consequently, to insulin resistance. Animal studies also showed that the deletion of afamin gene led to increased insulin sensitivity in rats (25). Koninger et al. found that serum afamin levels in patients with polycystic ovary syndrome was significantly higher than that of the normal control group, showing strong correlation with insulin sensitivity, and directly proportional to BMI (26). Potential mechanisms of the effect of afamin on insulin sensitivity may involve activation of the NLRP3 inflammasome and conversion of inactive caspase-1 precursors into active caspase-1, which mediates the release of IL-β. This model if further supported by the observation that afamin levels are directly proportional to the levels of hs-CRP (high-sensitivity C-reactive protein), an acute inflammatory protein (27). Studies show that a number of factors associated with diabetes, such as high glucose, adipokines, modified lipoproteins and free fatty acids may trigger CRP production by endothelial and smooth muscle cells and monocytes/macrophages. CRP is regulated by cytokines, and plays a critical role in T2DM by its action on pancreatic β-cells (28). Moreover, the serum level of high-sensitivity CRP are associated with β-cell dysfunction and insulin resistance (29).

A small-sample preliminary study also found that patients, requiring insulin to control blood sugar in late pregnancy, had higher concentrations of afamin compared to patients, whose blood glucose was managed with dietary modifications (30). A meta-analysis of the results of 8 prospective cohort studies involving 20136 samples, carried out by Kollerits B et al. (9), reported that afamin levels positively correlated with the HOMA-IR score of insulin resistance (β= 0.110, 95% CI 0.089–0.132, P = 1.37 × 1023), and were an independent prediction factor for 585 cases of diabetes. Adding serum afamin levels to the prediction model significantly improved its prediction accuracy. Every 10mg/L increase in Afamin level was associated with an increase in the incidence of type 2 diabetes (OR=1.19, 95% CI 1.12–1.26, P= 5.96×10^−8^). In our study, afamin levels of GDM patients were higher by an average of 69.16 mg/L, as compared to a non-GDM group. Furthermore, the results of multivariant logistic analysis indicated that afamin is a strong independent risk factor of GDM (P<0.001). Together with previous evidence, our results suggest that afamin can be used as an early biomarker for detection for abnormal glucose metabolism during pregnancy. We speculate that increased serum afamin levels may contribute to oxidative stress, insulin resistance and inflammation, ultimately leading to the development of GDM. This hypothesis is further strengthened by the observed evidence of chronic inflammation in GDM group, as indicated by the PLR and NLR levels. Platelet to lymphocyte and neutrophile to lymphocyte ratio (PLR and NLR respectively) are considered indicators of subclinical inflammation and useful predictive markers in prediabetes and diabetes mellitus. Studies show that PLR significantly decreases in prediabetes and early stages of diabetes, while NLR is significantly increased (31). Platelet functions can be evaluated by measuring platelet parameters such as platelet count, mean platelet volume (MPV), and platelet distribution width (PDW) (32). Gestational diabetes is a systemic disease, and the activation of platelets in diabetic patients often precedes the appearance of vascular pathological changes (33). The interaction between endothelial cells and platelets can be accompanied by the release of a variety of inflammatory factors, leading to the adhesion and migration of leukocytes (34–36). Platelet count and platelet-related parameters such as MPV have been studied in GDM, and it was found that MPV can effectively reflect blood glucose level (37). Our results indicated that the PLR ​​of GDM patients in the first trimester was significantly lower than that of the non-GDM group (P<0.001). This decrease coincided with the marked increase in NLR (P=0.011). However, as indicated by multivariant logistic regression analysis, only PLR may be considered a predictor and independent GDM risk factor in our study. Since the occurrence of GDM may be due to the activation of the immune system, lymphocyte-mediated immune system disorders may be the key pathophysiological cause of GDM, and platelets may play a particularly important role in the regulation of GDM immunity and inflammation. Therefore, the PLR ​​value is more effective than other platelet-related parameters in predicting GDM. Using PLR as an early marker of GDM also has the advantage of high efficiency, quick turn-around, and low price.

During pregnancy, blood lipid levels are physiologically elevated and the intestinal absorption of fat is increased to meet the needs of placental and fetal growth and development. This physiological increase generally does not cause adverse consequences and will gradually return to normal after the end of pregnancy (38). However, abnormally elevated blood lipids during pregnancy may lead to certain metabolic diseases like gestational diabetes and acute pancreatitis (39). At the same time, dyslipidemia can also induce oxidative stress in the body, aggravate vascular endothelial damage, and cause a series of pregnancy complications such as hypertension and cardiovascular disease (40). Recent studies have shown that elevated triglyceride (TG) levels may aggravate insulin resistance (40), lead to vascular endothelial cell damage (41) and correlate with the occurrence of GDM (42). In our study, hypertriglyceridemia in the first trimester was an independent risk factor of GDM, and demonstrated predictive significance. Early triglyceride screening may, thus, potentially help reduce the incidence of maternal and child complications.

In this study, we combined maternal age, early pregnancy parameters such as afamin level, TG, and PLR to investigate and build a predictive model for GDM. We concluded that the prediction accuracy of age combined with afamin level, TG, and PLR (AUC=0.748) is higher than any single factor, with AUC 0.653, 0.574, 0.622, 0.618 respectively, P<0.001. The prediction sensitivity, specificity, positive predictive value, and negative predictive value were 69.2%, 68.3%, 42.5%, and 86.2% respectively, with an accuracy rate of 70.2%. Overall, our results clearly indicate a good predictive value of the proposed model.

Additional potential risk factors, analyzed in this study, merit further investigation. BMI is an important indicator of human body fitness. Previous studies have shown that the probability of GDM in overweight pregnant women is significantly higher than that of normal pregnant women (43). However, there was no statistically significant difference in the pre-pregnancy body mass index between the two groups of pregnant women in this study (P>0.05), which needs to be further explored.

PAPP-A is a zinc-binding metalloproteinase, which is secreted by the placental trophoblast tissue in pregnant women. PAPP-A in maternal serum can be detected after 28 days of fertilization, and then it grows rapidly, but the growth rate is slower in the second trimester. At present, the biological function of the PAPP-A molecule during pregnancy is still unclear, but a number of studies have shown that low levels of PAPP-A are related to GDM (44, 45). In this study, the concentration of PAPP-A in the GDM group was lower than that in the control group. Although further multivariate analysis was not statistically significant, it still suggests that PAPP-A may be closely related to glucose metabolism, which merits further investigation.

In summary, the establishment of a simple, low-cost and effective predictive model for early prediction of GDM has clinical practical values and can provide a reliable basis for clinical decision-making. We also acknowledge that this study is a single-center study. Therefore, the proposed GDM prediction model needs further improvement. Additional in-depth prospective, larger-sample size-, and multi-center studies are needed to provide more powerful support for clinical diagnosis and treatment of GDM.

## Data Availability Statement

The raw data supporting the conclusions of this article will be made available by the authors, without undue reservation.

## Ethics Statement

The studies involving human participants were reviewed and approved by ethics committee of Fujian Maternity and Child Health Hospital, Affiliated Hospital of Fujian Medical University. The patients/participants provided their written informed consent to participate in this study.

## Author Contributions

XW conceived and designed the study. XZ, MX, and LZ were involved in literature search and data collection. LX and ZL analyzed the data. XW wrote the paper. JY and RX reviewed and edited the manuscript. All authors read and approved the final manuscript.

## Funding

This study is supported by Science and Technology Innovation Research Funding of Fujian Maternity and Child Health Hospital (YCXZ 18-20).

## Conflict of Interest

The authors declare that the research was conducted in the absence of any commercial or financial relationships that could be construed as a potential conflict of interest.

## Publisher’s Note

All claims expressed in this article are solely those of the authors and do not necessarily represent those of their affiliated organizations, or those of the publisher, the editors and the reviewers. Any product that may be evaluated in this article, or claim that may be made by its manufacturer, is not guaranteed or endorsed by the publisher.
